# Spatiotemporal of the Coupling Relationship between Ecosystem Services and Human Well-Being in Guanzhong Plain Urban Agglomeration

**DOI:** 10.3390/ijerph191912535

**Published:** 2022-10-01

**Authors:** Jianxiu Yang, Xing Ma, Xueyan Zhao, Wenqing Li

**Affiliations:** 1 College of Geography and Environment Science, Northwest Normal University, Lanzhou 730070, China; 2 Key Laboratory of Resource Environment and Sustainable Development of Oasis, Lanzhou 730070, China; 3 College of Earth and Environmental Sciences, Lanzhou University, Lanzhou 730000, China

**Keywords:** Guanzhong Plain urban agglomeration, ecosystem services, human well-being, InVEST model, coupling coordination degree

## Abstract

Understanding the complex relationship between ecosystem services and human well-being during the rapid development of urban agglomerations can promote the sustainable development of urban agglomerations. In this paper, the InVEST model and ArcGIS10.2 were used to analyze the spatial and temporal evolution characteristics of ecosystem services and human well-being in the Guanzhong Plain urban agglomeration. On this basis, the coupling coordination index is used to reveal the spatiotemporal coupling relationship between them. (1) From 2010 to 2018, the water conservation services, soil conservation services, and carbon sequestration services of the Guanzhong Plain urban agglomeration showed a fluctuating downward trend. The spatial differences of ecosystem services were significant. (2) From 2010 to 2018, human well-being in the Guanzhong Plain urban agglomeration showed a fluctuating downward trend, with a decrease of 17%, and regional differences tended to narrow. (3) The coupling coordination degree between ecosystem services and human well-being has slightly decreased while maintaining the basic coordination state. The results show that there was a significant relationship between the decline of ecosystem services and the rapid development of the Guanzhong Plain urban agglomeration, and policies should be classified according to the coupling coordination types of human well-being and ecosystem services to promote the sustainable development of urban agglomerations.

## 1. Introduction

Since the 21st century, along with the continuous expansion of global cities, growth in the intensity of human activities, and continuous increases in social demands and human demands for water, land, and energy have been increasing. These factors have intensified the exploitation of natural resources and severely damaged global ecosystem services [1]. Maintaining good ecosystem services in urban agglomerations and effectively improving local human well-being are hot topics for researchers. Therefore, the Millennium Ecosystem Assessment (MA), the Future Earth Program (GLP), and the 2030 Agenda for Sustainable Development, proposed by international organizations, have coordinated ecological and urbanization development as their goals. These international organizations have paid much attention to the relationship between ecosystem services and human well-being [2]. China’s urban agglomerations are in a stage of rapid development. The high-quality development of urban agglomerations, in addition to the high-level protection of ecological environments, and ultimately the improvement of human well-being, are realistic issues that need to be faced in the construction of urban agglomerations [3].

Ecosystem services are defined as the various benefits humans derive from ecosystems [4] and are now recognized as provisioning services, regulating services, cultural services, and supporting services [5], which aim to improve human well-being [6]. Since the 1990s, many scholars have conducted studies around the theory, method and practical application of ecosystem service supply assessment. Extensive assessments of ecosystem services have been conducted in different regions, scales, and types [5,6,7,8]. There are a variety of quantitative assessment methods for ecosystem services. Cotatanza [4] et al. used the value equivalent scale to estimate the total capital structure value of ecosystem services. The InVEST model was used to visualize the value of ecosystem services, and the sustainable and dynamic evaluation methods. These two methods are the most widely used [9]. In contrast, human well-being has no standard definition and remains a contested concept [10]. Now, it is generally believed that human well-being is multi-dimensional, and the selected indicators are not the same under different research scales [11]. Easily accessible statistical indicators are used in large-scale studies [12], such as the Human Development index (HDI) [11] and National Well-Being Index (NWI) [13], and comprehensive well-being evaluations combining quality-of-life and material conditions [13,14].

## 2. Literature Review and Research Framework

### 2.1. Literature Review

The core issue of sustainable science is to extend the research on ecosystem services to human well-being, and to study the relationship between them [15]. Yang Xueting et al. [16], Liu Ziwen et al. [17], Willis C., and Kosanic et al. [18,19] explored the relationship between ecosystem services and human well-being from the perspectives of provisioning services, regulation services, and cultural services. Li [20] and Wei et al. [21] studied the impact of the supply–demand ratio of ecosystem services and different types of supply–demand mismatch on human well-being. Robinson B.E. et al. [22] proposed land management strategies based on the dependence of farmers’ livelihoods on ecosystem services. Richard S. et al. [23] studied the impact of different decisions on human well-being from the community scale. Previous studies have shown that ecosystem services primarily play a bearing or constraint role in human well-being in terms of provisioning, regulation, culture, and support services [5], and the latter promotes or stresses ecosystem services as well as their functions through the differentiation of economic, social, and environmental well-being needs [24], thus forming a close bidirectional correlation between the two [25]. In the process of deepening geographical research into human–earth system coupling, ecosystem services and human well-being are increasingly closely interacting, and their correlation and coupling have gradually become the focus and frontier issues of current research [26].

In general, there are a lack of studies on the coupling relationship between ecosystem services and human well-being [26]. With the rapid development of urban agglomeration, many urban environmental problems have become increasingly prominent, and the ability of ecosystem to supply human well-being has been declining [27]. Assessing the coupling relationship between ecosystem service value and human well-being from the perspective of urban agglomerations can help cities maintain ecosystem service capacity and improve human well-being. This has important theoretical and practical significance for realizing the sustainable development of urban agglomerations.

### 2.2. Research Framework

**Figure 1:**
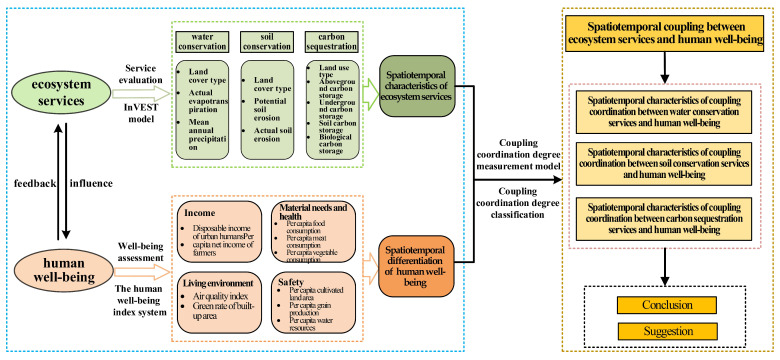
Research framework.

The Guanzhong Plain urban agglomeration is an ecologically sensitive area, located in an important area of ecological function. The special geographical location and complex topography aggravate the vulnerability of the regional ecological environment; the environmental capacity is close to its limit. As a typical Western urban agglomeration with the prominent contradiction of “human–land”, urban development and economic growth have intensified the waste of resources and resource constraints. At present, it is urgent to reveal the coupling relationship between human well-being and ecosystem services as well as to formulate a reasonable urban development strategy. Therefore, taking the Guanzhong Plain urban agglomeration as a research case, we used the InVEST model, coupling coordination degree, and other methods to analyze the coupling relationship and spatiotemporal evolution characteristics between ecosystem services and human well-being. In this way, the feedback of human well-being to ecosystem service changes and the well-being-driven effect of ecosystem service values were investigated. This provides a reference for the relationship between ecosystem services and well-being in the rapid urbanization of less developed regions around the world. The overall research framework is illustrated in Figure 1.

## 3. Materials and Methods

### 3.1. Study Area

**Figure 2:**
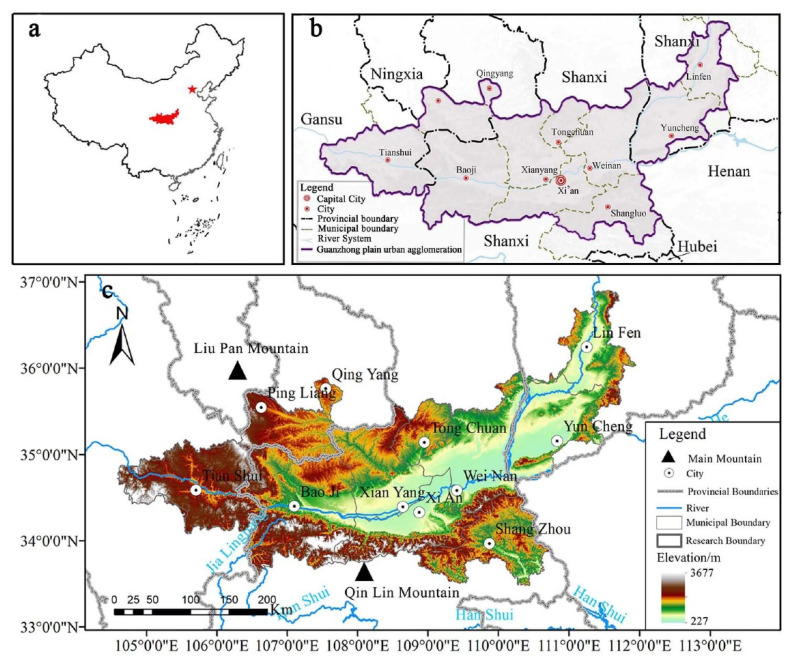
Overview of Guanzhong Plain urban agglomeration: (**a**) location in China; (**b**) administrative divisions; (**c**) elevation.

As the second largest urban agglomeration in western China, the Guanzhong Plain urban agglomeration is an important growth pole, leading the development of the western region, and an important gateway facing the central and eastern regions. It includes the Guanzhong region of Shaanxi Province and some cities in Shanxi and Gansu Provinces, with a total of 90 counties (cities and districts). With an area of 10.71 × 10^4^ km, and an average altitude of 400–3700 m (Figure 2), it possesses a temperate semi-humid monsoon climate. The rainfall decreases from west to east and from south to north. The regional geology and landforms are complex, with the mountains of the Southern Shaanxi and the Qinling Mountains to the south, the Loess Plateau to the north, and the Weihe River Lower Valley Plain in the middle, showing a basin topography with high surroundings and a low center. It is the core area of the middle reaches of the Yellow River Basin, and an important grain-producing area in China.

At the end of 2018, its permanent human population was 39.4853 million; its GDP was more than CNY 2 trillion, accounting for about 2.3% of the total GDP of China. However, the capacity of the natural ecological environment in this region is weak. Water resources are scarce, and groundwater overexploitation is prominent. The per capita water resources are less than one third that of the national average, and the spatial distribution of water resources is uneven. Water pollution in some sections of the Weihe River and Fenhe River Basin is serious. The massive mining of mineral resources has caused problems, such as soil erosion and soil pollution. It is necessary to strengthen the construction of ecological civilization and ecological environment protection in the future. The relationship between ecosystem services and human well-being is of great significance for solving the contradiction between ecological protection and economic construction in the Guanzhong Plain urban agglomeration.

### 3.2. Data Sources

This paper mainly includes meteorological data, soil data, land use data, and statistical yearbook data. The meteorological data are from resources and environment data cloud platform (https://www.resdc.cn/Default.aspx (accessed on 6 December 2021)). The potential evapotranspiration data are from the Global Drought Index and Potential Evapotranspiration (ET0) Climate Database V2. Soil data are from the World Soil Database. The land use data of 2010, 2015, and 2018 were from China Land Use/Land Cover Remote Sensing Monitoring Database with a resolution of 100 × 100 m. DEM data are from geospatial data cloud (http://www.gscloud.cn/ (accessed on 8 December 2021)). All raster data were reclassified and transformed into projections in GIS, the resolution was uniformly transformed into 100 m × 100 m, and the projection was uniformly transformed into Albers projection. The data for the assessment of human well-being are mainly from the 2010, 2015, and 2018 China Urban Statistical Yearbook, China County Statistical Yearbook, and the statistical yearbooks of 90 counties (districts) in the Guanzhong Plain urban agglomeration.

### 3.3. Research Methods

#### 3.3.1. Ecosystem Services

1. Water Yield model

As a typical water-scarce area, the Guanzhong area has rapidly increased the demand for water resources. The InVEST Water Yield model is used to calculate the water conservation services of the region, detailed in the following equations [28]:

$$Y_{\mathrm{xj}}=\left(1-\frac{{\mathrm{AET}}_{\mathrm{xj}}}{P_{x}}\right)\times P_{x}$$

$$\frac{{\mathrm{AET}}_{xj}}{P_{x}}=\frac{1\;+\omega_{x}R_{\mathrm{xj}}}{1+\omega_{x}R_{\mathrm{xj}}+\left(\frac{1}{R_{\mathrm{xj}}}\right)}$$

$$\omega_{x}=Z\frac{{\mathrm{AWC}}_{x}}{P_{x}}$$

$$R_{\mathrm{xj}}=\frac{K_{\mathrm{xj}}\times {\mathrm{ET}}_{x}}{P_{x}}$$

where $Y_{\mathrm{xj}}$ is the annual water volume (mm) of the land cover type j in pixel x. ${\mathrm{AET}}_{\mathrm{xj}}$ is the actual evapotranspiration (mm) of land cover type j in pixel x. $P_{x}$ is precipitation (mm) of pixel x. $\omega_{x}$ is to correct the ratio of annual vegetation available water and precipitation. $R_{\mathrm{xj}}$ is the dry coefficient. Z is Zhang’s coefficient, which is 30 [29] in this paper, according to previous studies. ${\mathrm{AWC}}_{x}$ is the effective soil water content (mm) of raster cell x. $K_{\mathrm{xj}}$ is the evapotranspiration coefficient of vegetation of land cover type j in pixel x. ${\;\mathrm{ET}}_{x}$ is the reference crop evapotranspiration.

2. Sediment Delivery Ratio (SDR) model

Soil erosion is a serious environmental problem faced by human beings that restricts the sustainable development of the global economy and society. Serious soil erosion can destroy land productivity, reduce biodiversity, threaten the regional ecological environment, and exacerbate poverty in mountainous areas. The Guanzhong Plain urban agglomeration is located in a fragile ecological environment. The soil erosion is very strong because of the combined action of natural and human factors. The Guanzhong Plain urban agglomeration is the key area of soil erosion research in the world. The soil retention in this area is calculated by the SDR Model in the InVEST model [30]:

$${\mathrm{SEDERT}}_{x}{=\mathrm{RKLS}}_{x}-{\mathrm{USLE}}_{x}$$

where ${\mathrm{SEDERT}}_{x}$ denotes soil conservation (t) of pixel x. ${\mathrm{RKLS}}_{x}$ and ${\mathrm{USLE}}_{x}$ denote potential soil erosion (t) and actual soil erosion (t), respectively.

$${\mathrm{RKLS}}_{x}{=R}_{x}\times K_{x}\times {\mathrm{LS}}_{x}$$

$${\mathrm{USLE}}_{x}{=R}_{x}\times K_{x}\times {\mathrm{LS}}_{x}\times C_{x}\times P_{x}$$

where $R_{x}\;$ is rainfall erosivity [MJ·mm/(hm·h·a)]. $K_{x}$ is the soil erodibility. ${\mathrm{LS}}_{x}$ is the slope length and slope factor. $C_{x}$ is the vegetation cover factor. $P_{x}$ is the management factor.

3. Carbon Storage and Sequestration model

We calculated carbon Sequestration services of Guanzhong Plain urban agglomeration through the Carbon Storage and Sequestration in InVEST model. Carbon sequestration mainly includes aboveground carbon sequestration, underground carbon sequestration, soil carbon sequestration, and biological carbon sequestration. The carbon density data are from the literature [31].

$$G_{C}{=G}_{\mathrm{above}}{+G}_{\mathrm{below}}{+G}_{\mathrm{dead}}{+G}_{\mathrm{soil}}$$

where $G_{C}$ is the total carbon sequestration of the ecosystem (t). $G_{\mathrm{above}}$ is the aboveground carbon sequestration (t). $G_{\mathrm{below}}$ is the underground partial carbon sequestration (t). $G_{\mathrm{dead}}$ is the carbon sequestration of dead organic matter (T). $G_{\mathrm{soil}}$ is the soil carbon sequestration (t).

#### 3.3.2. Human Well-Being Level

1. The construction and evaluation of human well-being indicators

According to the Millennium Ecosystem Assessment Report, well-being mainly refers to the material, spiritual, and health needs of human beings, including basic living materials, safety, health, good social relations, and freedom of choice as well as action [32]. Generally, easy-to-obtain statistical indicators are selected for large-scale evaluation [13]. Based on the well-being connotations and related research in the Millennium Ecosystem Assessment Report, in addition to the availability of data, this paper constructed a well-being evaluation index body for humans in the Guanzhong Plain urban agglomeration from four dimensions: income, material needs and health, living environment, and safety (Table 1).

Among them, the need for a good life are not confined to the need for food and clothing; they include those necessary for pursuing a high quality of life, living environment beauty, and happiness of a better life. Basic substances for a good life include residents’ income, purchasing power, and quality of life [32]. This paper will address residents’ income and consumption of grain, meat, and vegetables to measure the material needs of residents to farmers, as well as their health. Living environments are an important source of residents’ happiness. With the improvement of living standards, people pay more and more attention to the surrounding environment, and the degree of greenness and air quality are often the issues that are of most concern to urban residents [33]. Therefore, this paper includes an air quality index and the green rate of built-up areas in the evaluation indexes of human well-being [34]. In the arid region of northwest China, the ecological environment is fragile and the problems of soil erosion, desertification, and soil salinization are serious and threaten the livelihood and well-being of residents. Water resource security and food security are important factors affecting the well-being of residents [35,36]. Therefore, the per capita water resources, per capita cultivated land area, and per capita grain yield are used as the evaluation indexes of security.

In the evaluation, the entropy weight method was used to determine the weight of each index. The entropy weight method can determine the index weight according to the variation degree of the index value of each indicator. It is an objective weight method that avoids the deviations caused by human factors, gives full play to the advantages when determining the weights of many different indicators, and reflects the differences in the degree of fluctuation of different well-being dimensions [37]. Therefore, this paper first uses a range standardization method to standardize each index and determines the weight of each indicator via the entropy weight method. The well-being index of each county and district in the Guanzhong Plain urban agglomeration was then calculated via weighted summation. Finally, the overall well-being level of humans in the Guanzhong Plain urban agglomeration was evaluated through the average value of human well-being in each county.

2. Analysis of hot and cold spots

The spatial agglomeration degree of human well-being in Guanzhong Plain urban agglomeration was effectively identified by the cold–hot spot analysis.

$$G_{i}^{*}\left(d\right)=\frac{K_{\mathrm{xj}}\times \sum_{i=1}^{n}W_{\mathrm{ij}}\left(d\right)x_{i}}{\sum_{i=1}^{n}P_{x}}$$

where $W_{\mathrm{ij}}$ is weight. $x_{i}$ is the sample value of i. $G_{i}^{*}\left(d\right)$ is the degree used to effectively identify the spatial agglomeration degree of human well-being. If the value is positive, the area is a high-value agglomeration area of human well-being. Otherwise, it is a low-value agglomeration area.

#### 3.3.3. The Coupling Relationship between Ecosystem Services and Human Well-Being

In this paper, the coupling degree index was introduced to construct the coupling coordination degree [29,30,31,32] measurement model of urban ecosystem services and human well-being in the Guanzhong Plain. We studied the degree of interaction between ecosystem services and human well-being and characterized whether the functions are mutually promoting at high levels or constraining at low levels.

$$D=\sqrt{C\times T}$$

$$T_{Y}=\partial Y_{i}+\beta U_{i},\;T_{S}=\partial S_{i}+\beta U_{i},\;T_{G}=\partial G_{i}+\beta U_{i}$$

$$C_{Y}=2\times {\left\{\frac{Y_{i}\times U_{i}}{{\left(Y_{i}+U_{i}\right)}^{2}}\right\}}^{1/2},\;C_{S}=2\times {\left\{\frac{S_{i}\times U_{i}}{{\left(S_{i}+U_{i}\right)}^{2}}\right\}}^{1/2},\;C_{G}=2\times {\left\{\frac{G_{i}\times U_{i}}{{\left(G_{i}+U_{i}\right)}^{2}}\right\}}^{1/2}$$

where *D* is the degree of coupling coordination. *C* represents the coupling value and characterizes the degree of interaction between ecosystem services and human well-being, 0 ≤ *C* ≤ 1. *T* is a comprehensive evaluation index for the coordinated development of ecosystem services and human well-being, indicating the overall synergistic effect or the contribution of the two. $Y_{i}$, $S_{i}$, and $G_{i}\;$ are water conservation service, soil conservation service, and carbon sequestration service, respectively. $U_{i}$ is the human well-being index. ∂ and β are coefficients to be determined. Due to the coordinated development of ecosystem services and human well-being, both ∂ and β are set as 0.5.

Based on results of existing studies [12,38,39,40] and the actual situation of this study, the coupling coordination degree of “ecosystem services-human well-being” was classified into five types(Table 2).

## 4. Results

### 4.1. Spatial–Temporal Characteristics of Ecosystem Services

#### 4.1.1. Spatial–Temporal Characteristics of Water Conservation Services

**Figure 3:**
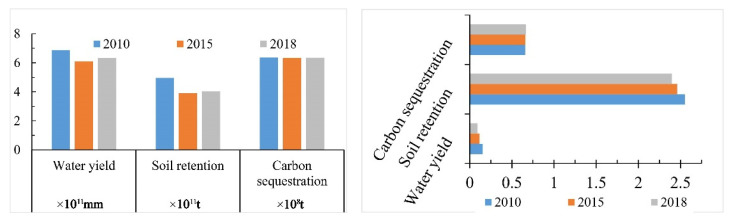
Total ecosystem services and coefficient of variation of ecosystem services.

From 2010 to 2018, the water content of the Guanzhong Plain urban agglomeration showed an overall fluctuating decreasing trend, from 6.88 × 10^11^ mm in 2010 to 6.34 × 10^11^ mm in 2018, a decrease of 7.8%. From 2010 to 2015, the water conservation of the Guanzhong Plain urban agglomeration significantly decreased, from 6.88 × 10^11^ mm to 6.11 × 10^11^ mm, a decrease of 11.3%. From 2015 to 2018, the annual water conservation slightly increased, from 6.11 × 10^11^ mm to 6.34 × 10^11^ mm, an increase of 3.8% (Figure 3). From 2010 to 2018, the coefficient of variation of water conservation services in the Guanzhong Plain urban agglomeration showed an overall downward trend, with a decrease of 16% (Figure 3). The aforementioned information indicates that the regional differences in water conservation services in this region were gradually narrowed.

Water conservation in the Guanzhong Plain urban agglomeration shows an overall distribution pattern of “high in the south and low in the north, decreasing from south to north”. The high-value areas of water conservation were mainly distributed in the northern foothills of the Qinling Mountains. The second highest value areas of water conservation were mainly distributed in the upper reaches of the Weihe River, the northwest of the Guanzhong Basin, and the east of the Guanzhong Plain urban agglomeration. The low-value areas of water conservation were mainly distributed in the northwest of the Guanzhong Plain urban agglomeration and the Longdong region of the Gansu Province. This is consistent with the spatial distribution pattern of rainfall in the Guanzhong Plain urban agglomeration. From 2010 to 2018, the high-value areas of water conservation in the Guanzhong Plain urban agglomeration showed an expansion trend from east to west, whereas the low-value areas tended to shrink. From 2010 to 2015, the high-value areas were mainly distributed in the southeast and south of the Guanzhong Basin, with an expanding trend. From 2015 to 2018, the high-value area continued to expand westward, basically forming a distribution pattern consistent with the ecological barrier zone in the Qinba Mountains area in the south of the Guanzhong Plain urban agglomeration.

#### 4.1.2. Spatial–Temporal Characteristics of Soil Conservation Services

From 2010 to 2018, the soil conservation in the Guanzhong Plain urban agglomeration showed a fluctuating downward trend from 4.96 × 10^11^ t to 4.05 × 10^11^ t, a decrease of 18.3%. From 2010 to 2015, the decreasing trend was significant, from 4.96 × 10^11^ t to 3.91 × 10^11^ t, a decrease of 21.2%. From 2015 to 2018, it slightly increased from 3.91 × 10^11^ t to 4.05 × 10^11^ t, an increase of 3.6%. The coefficient of variation of soil conservation services in the Guanzhong Plain urban agglomeration was decreased by 6.8% from 2010 to 2018. This indicates that the regional differences in soil conservation in the Guanzhong Plain urban agglomeration tended to narrow.

From 2010 to 2018, soil conservation in the the Guanzhong Plain urban agglomeration showed a spatial distribution of “high in the south and low in the north, high in the west and low in the east”. The high-value areas were mainly distributed in the northern foothills of Qinling and the southeast of Longlong in the south of the Guanzhong Plain urban agglomeration. The low-value areas were mainly distributed in the Weihe River Valley and the Fenhe River Valley. From the perspective of interannual changes, the overall spatial distribution of soil conservation did not change much. The overall soil conservation in 2018 was less than that in 2010. However, the soil conservation in the Longdong area, especially Tianshui, was more than that in 2010.

#### 4.1.3. Spatial–Temporal Characteristics of Carbon Sequestration Services

From 2010 to 2018, the carbon sequestration of Guanzhong Plain urban agglomeration was decreased from 6.36 × 10^8^ t to 6.35 × 10^8^ t, but the decrease was less than 1%. From 2010 to 2015, the carbon sequestration was decreased from 6.36 × 10^8^ t to 6.34 × 10^8^ t, a decrease of 0.3%. From 2015 to 2018, the carbon sequestration was increased from 6.34 × 10^8^ t to 63.5 × 10^8^ t. From 2010 to 2018, the coefficient of variation of carbon sequestration service in the Guanzhong Plain urban agglomeration showed an overall upward trend. This indicates that the regional difference in carbon sequestration service tended to increase, but the increase was small (0.92%). This indicates that the spatial variation of soil conservation was small during this period.

**Figure 4:**
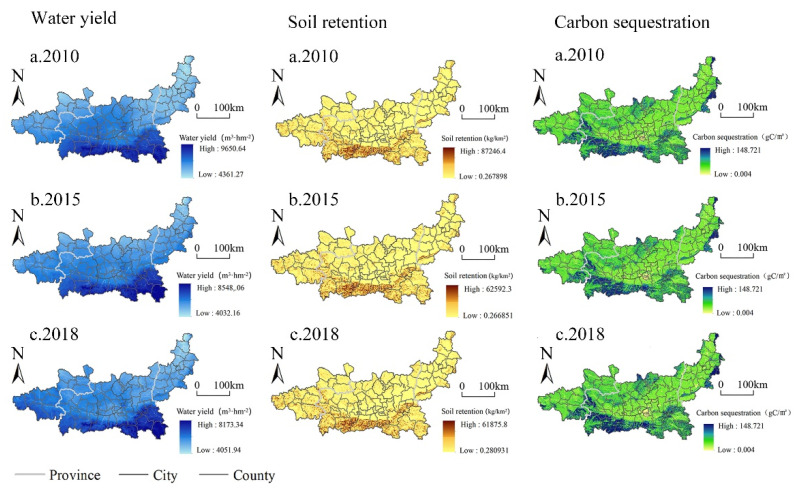
Spatial–temporal distribution of water conservation, soil conservation, and carbon sequestration in Guanzhong Plain urban agglomeration from 2010 to 2018.

From 2010 to 2018, the spatial distribution of carbon sequestration in the Guanzhong Plain urban agglomeration was “high in southwest China and low in northeast China”. The high-value areas were mainly distributed in the northern part of the Guanzhong Plain urban agglomeration, the northern part of Guanzhong Basin, the interlaced zone between the Liupan Mountains and the Guanzhong Basin, and the interlaced zone between the Taihang Mountains and the Jinshan Basin. The low-value areas were mainly distributed in the Weihe River Valley and the Fenhe River Valley. From the perspective of inter-annual variation, the overall spatial distribution of carbon sequestration did not change much. However, the carbon sequestration in 2018 was less than that in 2010 (Figure 4).

### 4.2. Spatial-Temporal Differentiation of Human Well-Being

**Figure 5:**
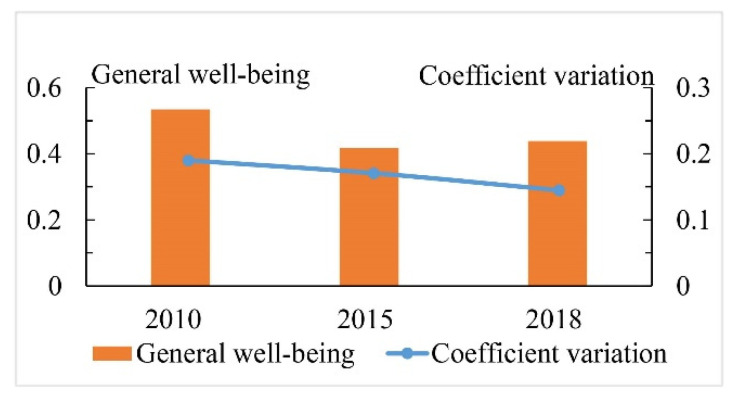
Changing trend of human well-being in Guanzhong Plain urban agglomeration from 2010 to 2018.

From 2010 to 2018, the comprehensive human well-being in the Guanzhong Plain urban agglomeration showed a fluctuating downward trend, from 0.53 in 2010 to 0.44 in 2018, a decrease of 17%. From 2010 to 2015, the human well-being showed a downward trend, from 0.53 to 0.42, a decrease of 21%. From 2015 to 2018, the human well-being showed a slow upward trend, from 0.42 to 0.44, an increase of 5%. The coefficient of variation of human well-being in the Guanzhong Plain urban agglomeration showed a downward trend, from 0.19 in 2010 to 0.14 in 2018, a decrease of 26% (Figure 5). This indicates that the regional differences in human well-being in the Guanzhong Plain urban agglomeration tended to narrow.

**Figure 6:**
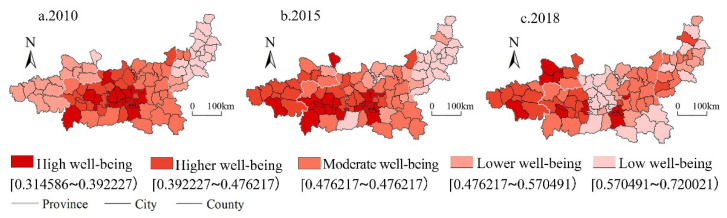
Spatial distribution of human well-being in Guanzhong Plain urban agglomeration from 2010 to 2018.

We classified the human well-being into five levels: high well-being, higher well-being, moderate well-being, lower well-being, and low well-being, by natural break point method (Figure 6). From 2010 to 2018, the overall human well-being in Guanzhong Plain urban agglomeration showed the spatial distribution of “high in the west and low in the east, high in the middle and low in the surrounding areas”. In 2010, the high well-being areas were mainly concentrated in the urban functional developed areas of the Weihe River Valley and surrounding areas, with a stepped distribution from high to low from the center to surrounding areas. From 2010 to 2015, the urban functional developed areas and surrounding high well-being areas in the Weihe Valley expanded westward. A total of 10.5% of the counties and districts were transformed from medium- and low-level areas to high well-being areas, forming a stepped distribution of high-level districts with Xi’an and Baoji as the dual cores in the urban functional developed areas of the Wei River Valley. Therefore, high well-being generally shows an expansion trend. Low well-being areas expand eastward from Yuncheng City and Linfen City in the interlaced area of the Taihang Mountains and the Shanxi-Shaanxi Basin, but the overall change was not significant. From 2015 to 2018, 16.7% of counties and districts were transferred from low-level and high-level well-being areas to medium-level and high-level well-being areas. The regional difference in human well-being was further narrowed. In addition, high well-being areas expanded to the northwest on the basis of the previous stage. A total of 7.8% of the counties and districts were transferred to low-level areas. Xianyang City and Zhouzhi County in the central part of the study area, and Shangluo City in the southeast of Guanzhong Basin were transferred from moderate well-being areas and high well-being areas to low-level areas. There was a slight shrinking of high well-being areas, and the distribution was dispersed.

**Figure 7:**
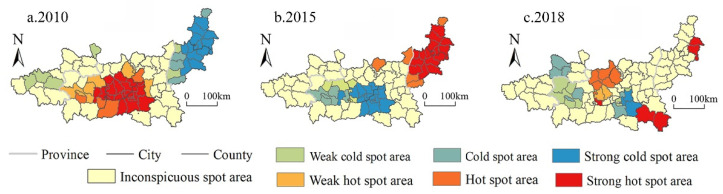
Spatial agglomeration characteristics of human well-being in Guanzhong Plain urban agglomeration from 2010 to 2018.

From 2010 to 2018, the spatial relationship of human well-being in the urban agglomeration in the Guanzhong area showed a “double contraction” trend, that is, the agglomeration of high-level and low-level areas of human well-being in the Guanzhong Plain urban agglomeration tended to weaken (Figure 7). From 2010 to 2015, the spatial relationship of human well-being showed a trend of “thermal contraction and cold expansion”. The hot spots were mainly distributed in Xi’an, Xianyang, and eastern Baoji in the Weihe Valley. The cold spot area expanded from Linfen City and Yuncheng City in the intersection of the Taihang Mountains and the Shanxi-Shaanxi Basin to the Shanxi-Shaanxi junction area. From 2015 to 2018, the hot area and cold spot area both tended to shrink, and the hot area continued to shrink on the basis of the previous stage. Baoji City and Pingliang City at the border of Guanzhong Basin and the Longdong region were the core of the sub-hot area. The cold spot area turns from Linfen and Yuncheng in the Shanxi-Shaanxi border area to the northern part of the Guanzhong Basin.

### 4.3. Spatiotemporal Coupling between Ecosystem Services and Human Well-Being

#### 4.3.1. Spatial–Temporal Characteristics of Coupling Coordination between Water Conservation Services and Human Well-Being

**Figure 8:**
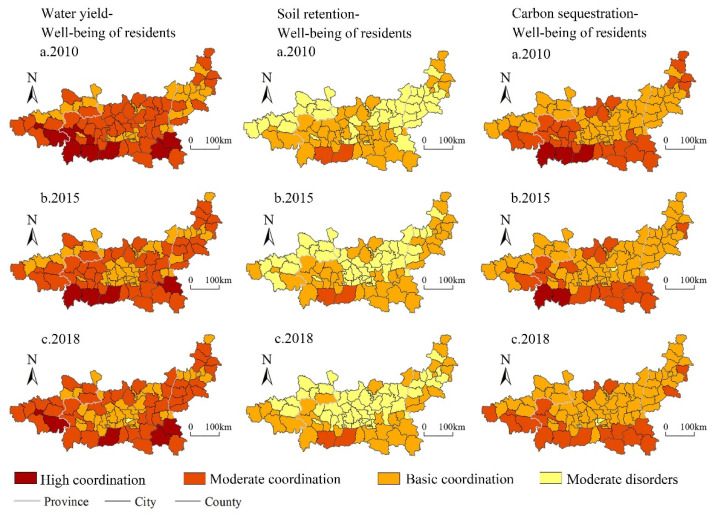
Spatiotemporal coupling patterns of water conservation, soil conservation, carbon sequestration, and human well-being in Guanzhong Plain urban agglomeration from 2010 to 2018.

The coupling coordination degree of “water conservation and human well-being” was significantly higher than that of “soil conservation services and human well-being” and “carbon sequestration services and human well-being” in the Guanzhong Plain urban agglomeration during the same period (Figure 8).

In the spatial dimension, the coupling coordination degree of “water conservation and human well-being” gradually spread out from the core of urban functional developed areas of central cities, with a significant spatial distribution of “high around and low in the middle”. The high-value areas were mainly distributed in the ecological protection areas with the Loess Plateau ecological barrier zone and the Qinba mountain ecological barrier zone, whereas the low-value areas were mainly concentrated in the central urban functional developed areas. The low-value areas were centered on the urban functional developed areas, and gradually expanded in the form of circle, and its influence scope was gradually enlarged.

In the temporal dimension, the overall coupling coordination degree of “water conservation and human well-being” was decreased from moderate coordination to basic coordination. The average level of coupling coordination degree was decreased from 0.64 to 0.60. In 2010, the coupling coordination degree was [0.46, 0.89], and the coupling coordination types mainly included basic coordination, moderate coordination, and high coordination, accounting for 41.11%, 51.11%, and 7.78%, respectively. The overall level was relatively high, and most of them were moderate coordination. In 2015, the coupling coordination degree was [0.42, 0.85]. In this period, the coupling coordination types of “water conservation and human well-being” were still basic coordination, moderate coordination, and high coordination, accounting for 48.9%, 46.6%, and 4.5%, respectively. The overall level was lower than that of 2005. In addition, the proportion of high coordination was lower than that of 2015. In 2018, the coupling coordination degree of “water conservation and human well-being” was between [0.39, 0.84]. The coupling coordination types in this period were moderate imbalance, basic coordination, moderate coordination, and high coordination, accounting for 1.1%, 47.8%, 46.7%, and 4.4%, respectively. The overall coordination level was moderate coordination.

The results show that the coupling coordination degree of “water conservation services and human well-being” of all districts and counties in the Guanzhong Plain urban agglomeration has a downward trend from moderate coordination to basic coordination.

#### 4.3.2. Spatial–Temporal Characteristics of Coupling Coordination between Soil Conservation Services and Human Well-Being

The coupling coordination degree of “soil conservation services and human well-being” showed a fluctuation pattern of “increase first and then decrease”, and the overall level was low.

In the spatial dimension, the low-value areas of coupling coordination degree of “soil conservation services and human well-being” in the Guanzhong Plain urban agglomeration were mainly distributed in the northern and central Guanzhong Plain urban functional developed areas, with the overall spatial distribution of “high in the south and low in the north”. The high-value areas were concentrated in the northern Qinba mountain area, and the low-value areas were distributed in the northern Guanzhong Basin bounded by the Weihe River Valley.

In the temporal dimension, the coupling coordination degree of “soil conservation services and human well-being” in the Guanzhong Plain urban agglomeration showed a gradual decline from basic coordination to moderate imbalance. The average level of coupling coordination degree was decreased from 0.42 to 0.4. In 2010, the degree of coupling coordination was [0.30, 0.66]. The types of coupling coordination were mainly from basic coordination to moderate imbalance, basic coordination, and moderate coordination, accounting for 46.67%, 51.11%, and 2.22%, respectively. The overall level was relatively high and was in the state of basic coordination. In 2015, the coupling coordination degree was [0.32, 0.69]. The coupling coordination types of “soil conservation services and human well-being” were moderate imbalance, basic coordination, and moderate coordination, accounting for 51.11%, 46.67%, and 2.22%, respectively. The proportion of moderate coordination was decreased. The overall level was lower than that in 2010. In 2018, the coupling coordination degree of “soil conservation services-human well-being” in Guanzhong plain urban agglomeration was [0.28, 0.65]. The types of coupling coordination degree of “soil conservation services and human well-being” were moderate imbalance, basic coordination, and moderate coordination, accounting for 58.89%, 38.89%, and 2.22%, respectively. The overall level was moderate imbalance.

In general, the coupling coordination degree of “soil conservation services and human well-being” in the Guanzhong Plain urban agglomeration showed a fluctuating downward trend from basic coordination to moderate imbalance. The regional differentiation of coupling coordination degree did not change, but different regions showed different evolution trends. The overall coupling coordination degree in the northern Weihe River Valley continued to decline. However, the regional ecosystem services and human well-being in the southern Qinba Mountain and the southern Longdong Loess Plateau mutually promoted each other, orderly and benign high-level coupling coordination.

#### 4.3.3. Spatial–Temporal Characteristics of Coupling Coordination between Carbon Sequestration Services and Human Well-Being

The coupling coordination degree of “carbon sequestration services and human well-being” in the Guanzhong Plain urban agglomeration was poor. The overall coupling coordination was at a low level. The carbon sequestration services were relatively weakened.

In the spatial dimension, the coupling coordination degree of “carbon sequestration services and human well-being” in the Guanzhong Plain urban agglomeration showed a significant spatial distribution of “high in the south and low in the north, and high in the west and low in the east”. The high-value areas were concentrated in the northern Qinba Mountains in the south. The low-value areas were mainly distributed in the areas with developed urban functions, such as Xi’an and Xianyang in the Guanzhong Plain, the Loess Plateau of Longdong, and the northeastern and western parts of the Guanzhong Plain.

In the temporal dimension, the coupling coordination degree of “carbon sequestration services and human well-being” in the Guanzhong Plain urban agglomeration showed a gradual declining trend from basic coordination to moderate imbalance. The average level of coupling coordination degree was decreased from 0.59 to 0.54. In 2010, the coupling coordination degree was [0.44, 0.86]. The coupling coordination types were basic coordination, moderate coordination, and high coordination, accounting for 67.78%, 28.89%, and 3.33%, respectively. The overall level was relatively high as basic coordination. In 2015, the coupling coordination degree was [0.39, 0.82]. The high-value areas in the southeast of the Guanzhong Plain urban agglomeration expanded to Fenhe Valley with Shangluo as the core, whereas the high-value areas in the west gradually shrunk with Tianshui in the southeast of the Longhe Plain as the core. The coupling coordination types of “carbon sequestration services and human well-being” were moderate imbalance, basic coordination, moderate coordination, and high coordination, accounting for 1.1%, 78.89%, 17.78%, and 2.22%, respectively. The overall level was lower than that in 2010. In 2018, the coupling coordination degree of “carbon sequestration services and human well-being” in the Guanzhong plain urban agglomeration was [0.36, 0.77]. The coupling coordination degree of “carbon sequestration services and human well-being” was promoted. The “Shangluo-Xi’an” cluster gradually shrunk, and the “Tianshui-Baoji” cluster shifted to the west while shrinking. The low-value area was gradually expanding outward with the urban functional developed areas in the Guanzhong Plain, especially the urban area of Xi’an as the core. There are three types in this period: moderate imbalance, basic coordination, and moderate coordination, accounting for 3.33%, 76.67%, and 20%, respectively. The overall level was basic coordination.

**Figure 9:**
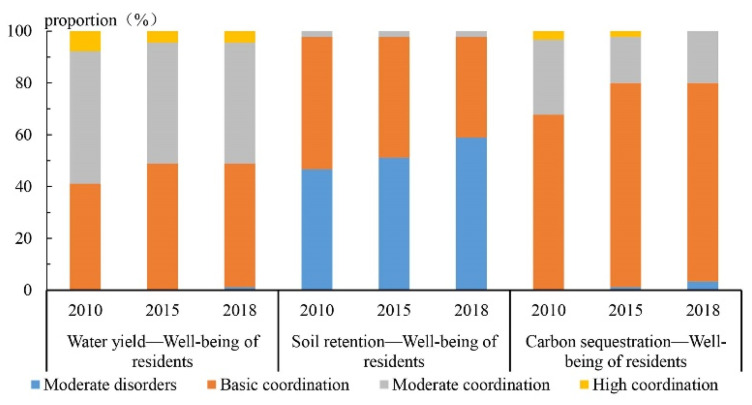
Changes of coupling coordination degree between ecosystem services and human well-being in Guanzhong Plain urban agglomeration from 2010 to 2018.

In general, the coupling coordination degree of “carbon sequestration services and human well-being” in districts and counties of the Guanzhong Plain urban agglomeration maintained the basic coordination state, whereas the overall level slightly decreased. The overall ordered and coordinated coupling coordination degree of “carbon sequestration services and human well-being” was degraded, and the number of high-value areas decreased (Figure 9).

## 5. Discussion

As an ecologically sensitive area, the Guanzhong Plain urban agglomeration has a relatively fragile ecosystem. With the rapid development of urbanization, population density is increasing, industrial structures are changing, and construction land is expanding. These aspects lead to resource consumption and environmental pollution, which make the ecosystem face more severe pressure. This paper found that the three types of ecosystem services in the Guanzhong Plain urban agglomeration showed a downward trend from 2010 to 2018. This is closely related to the long-term human activities in the Guanzhong Plain. In the process of urban agglomeration construction, urban expansion encroaching on cultivated land space is serious. The vegetation coverage around the city has been severely damaged, resulting in serious pressure on the ecosystem. Wu Jiansheng et al. [41] found that the land carrying capacity in Guanzhong was decreasing, and that the land ecological deficit was increasing year by year. The sustainable development situation was not optimistic. Second, it is greatly influenced by geographical conditions and climatic factors. The most direct influencing factors are the interannual variation in precipitation and vegetation coverage. Severe soil and water loss as well as extreme water shortages in the Guanzhong Plain led to a reduction in vegetation. This greatly restricted the large-area coverage of vegetation. From 2010 to 2018, the area of forest land in the region decreased by 5.21%, the area of grassland decreased by 11.3%, and the annual rainfall decreased by 8.2%. These led to a decrease in the demand for ecosystem services. Bai Yujuan et al. [42] found that plants in the Loess Plateau mainly rely on precipitation for growth. However, there were serious water shortages in this region, and the vegetation coverage was low. Therefore, ecosystem services were declining.

From 2010 to 2018, human well-being in the Guanzhong Plain urban agglomeration showed a fluctuating downward trend, with a decrease of 17%. From 2010 to 2015, human well-being showed a downward trend. From 2015 to 2018, human well-being showed an upward trend, but did not return to the level seen in 2010. The human well-being indicators in this paper are mainly constructed from four dimensions: income, material needs and health, living environment, and safety. From 2010 to 2015, income as well as material needs and health in the Guanzhong Plain urban agglomeration increased, but living environment and safety significantly decreased. Air quality, urban area, the area of cultivated land per capita, green area, and per capita grain output all showed a downward trend. In the construction of the urban agglomeration the population increased, arable land was occupied, and per capita cultivated land decreased. In addition, many farmers began to work in cities, leaving their farmland abandoned. The air pollution in the urban development is relatively serious. Xi’an and Xianyang were the most concentrated areas of atmospheric pollution. As a result, human well-being is declining. The implementation of the national food security strategy and targeted poverty alleviation strategy proposed at the 2013 Central Rural Work Conference has greatly increased humans’ income and improved farmland protection. In addition, Yang Ke et al. found that although the annual average PM2.5 concentration in the Guanzhong Plain urban agglomeration showed an overall downward trend from 2015 to 2019, it still exceeded China’s air quality Level II (35 μg/m^3^) [43]. Therefore, human well-being recovered from 2015 to 2018 but did not reach its initial level.

Human well-being is strongly dependent on the services provided by well-functioning ecosystems. Changes in the ecological functioning of systems can have direct or indirect effects on human well-being. The sustainable development of the Guanzhong Plain can only be ensured by realizing the coordinated development of human well-being and ecological environments. From 2010 to 2018, the level of coupling coordination between ecosystem services and human well-being in the Guanzhong Plain urban agglomeration showed a downward trend. Moreover, the spatial–temporal coupling relationship between ecosystem services and human well-being was lower in the developed urban areas and higher in the ecological protection areas dominated by the Loess Plateau ecological barrier zone and the Qinba Mountains ecological barrier zone. The results show that the disorderly expansion of the Guanzhong Plain urban agglomeration and the decrease in ecosystem services were significantly related to the rapid development of the Guanzhong Plain urban agglomeration. In the central region of urban functional developed areas, the economy has rapidly developed, and the land types dramatically changed in the urban agglomeration. The construction land occupied other types of land, especially in the urban fringe area where the ecological stress was the most serious. The main source of new construction land in urban expansion was cultivated land. Therefore, the coupling coordination degree between ecosystem services and human well-being was low. In the ecological protection areas dominated by the ecological barrier belt of the Loess Plateau and the Qinba Mountains, the quality requirements of the ecological environment are constantly improving, and the ecological environment is relatively good. The support of ecological compensation and poverty alleviation policies in the protection zones has significantly increased human well-being; human well-being was rapidly promoted in urban functionally developed areas. Therefore, the regional ecosystem services are highly coordinated with human well-being.

This paper studies the complex relationship between ecosystem services and human well-being during the rapid development of urban agglomerations, which provides a basis for the regional sustainable development of urban agglomerations in the arid region of northwest China. However, there are also some shortcomings. For example, in the process of assessing ecosystem services, some parameters in the InVEST model are based on the model user manual and the results of previous studies. In the future, field monitoring will be conducted to enhance the accuracy of the parameters in the study area, so as to improve the accuracy of ecosystem services’ assessment results. This paper takes counties as the research units, and we focus on the macro level of ecosystem services as well as the welfare of the mutual influence between them; however, we found that the accessibility of medical facilities, the development of traffic, and a balanced diet are also important factors influencing human well-being. Due to these data, our paper positions itself within the research as a study on welfare into the future.

## 6. Conclusions and Suggestion

### 6.1. Conclusions

Understanding the complex relationship between ecosystem services and human well-being can promote the sustainable development of urban agglomerations. Taking the Guanzhong Plain urban agglomeration as a case area, we used the InVEST model and the coupling coordination model to analyze the spatial–temporal pattern and the coupling coordination degree of ecosystem services and human well-being, based on the water conservation, soil conservation, and carbon sequestration services of the Guanzhong Plain urban agglomeration in 2010, 2015, and 2018. We have the following conclusions:

(1) From 2010 to 2018, three types of ecosystem services in the Guanzhong Plain urban agglomeration showed a downward trend. The amount of water conservation services showed a fluctuating downward trend, with a decrease of 7.8%. It showed a spatial distribution of “high in the south and low in the north, decreasing from south to north”. The amount of soil conservation services showed a fluctuating downward trend, with a decrease of 18.3%. It showed a spatial distribution of “higher in the south and lower in the north, higher in the west and lower in the east”. The carbon sequestration services showed a fluctuating downward trend, with a decrease of less than 1%. It showed a spatial distribution of “high in the southwest and low in the northeast”, and the regional differences tended to expand.
(2) From 2010 to 2018, human well-being in the Guanzhong Plain urban agglomeration showed a fluctuating downward trend, with a decrease of 17%. It showed a spatial distribution of “high in the middle and low around”. Regional differences tended to narrow, and the agglomeration of high-level and low-level areas of human well-being tended to weaken.
(3) From 2010 to 2018, the coupling coordination degree between ecosystem services and human well-being in the Guanzhong Plain urban agglomeration showed a downward trend. The coupling coordination degree of “water conservation services and human well-being” showed a spatial distribution of “high around and low in the middle”. The overall coordination decreased from moderate to basic coordination. The coupling coordination degree of “soil conservation services-human well-being” showed a distribution of “high in the south and low in the north”. Different regions showed different evolution trends. The overall trend decreased from basic coordination to moderate imbalance. The coupling coordination degree of “carbon sequestration services and human well-being” showed a significant distribution of “higher in the south and lower in the north, higher in the west and lower in the east”. The overall level was slightly degraded while maintaining the basic coordination state.

### 6.2. Suggestion

Based on the above conclusions, we classified and implemented policies based on the coupling coordination types of human well-being and ecosystem services to promote the sustainable development of the Guanzhong Plain urban agglomeration.

The following are suggestions for areas with lagging water conservation services. In the construction of future urban agglomerations, we should give priority to water resource protection and water conservation, optimize the distribution pattern and efficiency of water resources, and optimize the urban spatial layout, industrial structure, and population size with the carrying capacity of water resources. The government should adhere to the bottom line for the sustainable development of the Guanzhong Plain, Qinling, and Wei River as well as Fenhe River basin ecologically sensitive areas, such as water conservation functions, and speed up the development and protection of ecological sources in the urban functionally developed areas of the Wei River valley. Improving the security of water supplies and water conservation can expand the space of city development and promote human well-being. For the areas with lagging soil conservation services, it is necessary to coordinate soil and water conservation with urban agglomeration construction and regional high-quality development in future urban agglomeration construction processes. We should implement the ecological red line and return sloping land above 25° to forest (grass), soil and water conservation, and the comprehensive treatment of soil and water loss to promote urban greening construction and improve urban livability. In addition, it should be noted that the conversion of farmland into forest is not suitable for all regions, especially the Loess Plateau region in the north, where water resources are limited. Therefore, ecological conversion should be arranged according to scientific laws. For areas with lagging carbon sequestration services, it is necessary to change the development ideas and the methods of economic growth, accelerate industrial structure optimization, reduce the degree of interference with ecological systems, strictly control energy-intensive and highly polluting industries of low benefit, set up green industry systems, strengthen the protection and construction of forest ecological systems, and promote the mutual promotion of urban agglomeration construction as well as ecosystem protection and restoration, thereby achieving the coordinated development of ecosystem services and human well-being. By regulating ecosystem services to improve air quality, we can improve the health of urban humans and the overall ecological environment. Ecological corridors are unevenly distributed in the urban agglomeration, and the main ecological sources and corridors are concentrated in the southern Qinling region of the urban agglomeration. In the future development planning of the urban agglomeration, we should pay more attention to the ecological construction of the Qinling National Park and accelerate the ecological corridor.

Ecosystem services and human well-being are important extensions of sustainable development theory. The carrying capacities of regional environments are important limiting factors for the coordinated development of urban agglomerations. The services provided by good ecosystems within urban agglomerations can effectively improve local human well-being. At present, China’s urban agglomerations are in a stage of rapid development. To improve the spatial utilization efficiency of urban agglomerations, we should consider many factors, such as industries, spatial layouts, and ecological corridors, solve the environmental protection problems in the past single-city development mode stage, and coordinate the relationship between ecological protection and social as well as economic development. The coordination between the related goals of improving human well-being in urban agglomerations and the status quo of urban ecological protection is not only conducive to guiding the rational spatial layouts of urban agglomerations, but also crucial to improving internal ecological joint prevention and control ability in addition to the sustainable development of urban agglomerations. The construction of ecosystem security patterns is of great significance for the comprehensive management of hills, water, forests, fields, lakes, and grass, the formulation of multi-level ecological security policies, and the promotion of the resilience of urban agglomerations as well as the sustainable development goals of human well-being. In future research, it will be necessary to combine economic and social development factors, explore the internal source construction of urban agglomerations under the balance of the supply and demand of ecosystem services, strengthen the comprehensive management of urban agglomerations, and promote the regional integration in addition to high-quality development of urban agglomerations.

## Figures and Tables

**Table 1:** The human well-being index system of humans in Guanzhong Plain urban agglomeration.

The Target Layer	Level Indicators	The Secondary Indicators	Weight
Human well-being	Income	Disposable income of urban humans	19.00%
		Per capita net income of farmers	17.00%
	Material needs and health	Per capita food consumption	13.00%
		Per capita meat consumption	7.00%
		Per capita vegetable consumption	9.00%
	Living environment	Air quality index	15.00%
		Green rate of built-up area	1.00%
	Safety	Per capita cultivated land area	5.00%
		Per capita grain production	3.00%
		Per capita water resources	11.00%

**Table 2:** Types of coupling coordination degree between ecosystem services and human well-being.

Coupling Coordination Degree	Coupling Coordination Type	Characteristics
D ∈ (0, 0.2]	Serious imbalance	Ecosystem services and human well-being are mutually restricted. Excessive and disorderly development of urban agglomeration has seriously squeezed ecological space. This is contrary to human well-being.
D ∈ (0.2, 0.4]	Moderate imbalance	There are certain constraints on ecosystem services and human well-being. The ecological problems arising from the construction of urban agglomeration have become prominent, with a negative impact on human well-being.
D ∈ (0.4, 0.6]	Basic coordination	The relationship between ecosystem services and human well-being is basically harmonious. The construction of urban agglomeration can maintain healthy development.
D ∈ (0.6, 0.8]	Moderate coordination	Ecosystem services and human well-being can promote each other at a high level, and the construction of urban agglomerations can healthy develop.
D ∈ (0.8, 1.0]	High coordination	Ecosystem services and human well-being mutually promote each other at a high level, and urban agglomeration construction is developing in an orderly manner.
